# Beneficial effects of imagination of successful action after an actual error on baseline performances in non-expert young tennis players

**DOI:** 10.1007/s00426-024-02051-7

**Published:** 2024-11-16

**Authors:** Robin Nicolas, Robbin Carien, Younès Ouarti, Dominique Laurent

**Affiliations:** 1 Faculté des Sciences du Sport; Laboratoire “Adaptation au Climat Tropical, Exercice & Santé”, Université des Antilles, campus de fouillole, Pointe à Pitre, 97159 France; 2 Laboratoire IRISSE, UFR des Sciences de l’Homme et de l’Environnement, Département STAPS, Université de la Réunion, Le Tampon, France

## Abstract

The aim of this study was to evaluate the effects of motor imagery (MI: imagining the success or failure of a forehand or backhand shot) training according to an internal visual modality centred on the movement and the target to be reached on tennis performance. 66 young (M_age_ = 12.1 years) players were randomly divided into three groups: control, failure MI or success MI, and performed 3 experimental phases. The pre-test consisted of performing 6 blocks of 5 forehand and backhand groundstrokes (sent randomly by a ball launcher towards the baseline) and a super tie-break. The acquisition phase consisted of 12 sessions, each including a standardized warm-up followed by 15 min of background rally in pairs. The participants of the MI group were instructed, after unprovoked errors on their part, to imagine performing the previous shot correctly (positive MI) or missing (failure MI). The post-test was identical to the pre-test. The efficiency score of shots and the number of errors committed at the pre- and post-test served as dependent variables. The results of this study indicate that participants in the success MI group performed better than the control and failure MI groups at post-test. The success MI, performed after errors, has positive effects on the quality of the shot and reduces the number of unforced errors of tennis players, while failure MI induces negative outcomes. The use of success MI, integrated in training session, is recommended.

Tennis is a sport, which consists of hitting a ball, with a racket (Lees, 2003), in order to send it once more into the court than your opponent (Martin, 2018). Playing tennis requires physiological, technical, tactical, cognitive, perceptual-motor and mental skills (Cece et al., 2020), precision in the motor actions performed as well as anticipation-coincidence skills (Akpinar et al., 2012). This is why learning to play tennis requires a significant amount of practice sessions and rehearsals, which are generally spread over several years (Robin & Dominique, 2022).

In order to accelerate the learning speed and optimize the performance of tennis players, Corrado et al. (2020) suggested using mental training and integrating them into programs and training sessions. The mental aspect of tennis performance represents a central concern for players and coaches (Dominique et al., 2021). In a recent review on racquet sports, Cece et al. (2020) showed that the most frequently used mental skill was motor imagery (MI).

MI can be defined as a conscious process during which individuals internally simulate a motor action without actually carrying it out (Robin et al., 2006). It is a dynamic state during which the representation of a specific motor action is reactivated in the brain in the absence of real movement (Decety & Jeannerod, 1995). Many authors revealed that real execution and MI are functionally similar (Decety, 1996; Rice & Rubin, 2009). Indeed, numerous research studies based on experimental paradigms such as the measurement of cerebral, electro-myographic or neurovegetative activities have highlighted similarities between these two types of practice (e.g., Batula et al., 2017; Boschker, 2001; Collet et al., 1999) that can explain the positive effects of MI on learning, motor performance and rehabilitation (Guillot et al., 2012). Many studies reported the use of MI training in sport context (Fourkas et al., 2008; Mandolesi et al., 2023; Montuori et al., 2018; Zhang et al., 2019). For example, MI is a mental strategy that is frequently used by tennis players and coaches, in various ways such as increasing self-confidence, focus, self-efficacy, concentration or reducing anxiety (i.e., motivational functions) and mental rehearsing of strategies or specific sport skills (i.e., cognitive functions) such as imagining groundstroke shot (for review see Robin & Dominique, 2022). Recent studies found that MI intervention can increase athletes’ self-confidence in performing motor task (e.g., Prastyawan et al., 2023). Other research work has also shown beneficial effects of MI on motor learning (e.g., Cherappurath & Elayaraja, 2017; Guillot et al., 2012; Robin et al., 2023) and performance (e.g., Cherappurath et al., 2020; Dominique et al., 2021; Fekih et al., 2020) of technical gestures. For example, Guillot et al. (2013) showed that young tennis players obtained higher performances (i.e., precision, speed and percentage of success of the first serve balls) after sessions combining real practice and MI than in a control condition (i.e., physical practice only). Similar results were obtained by Robin et al. (2007) who observed beneficial effects of practicing internal visual imagery (i.e., imagining seeing the changes resulting from one or more actions from the point of view of the performer as if one were seeing themselves from his own eyes) on the accuracy of service returns. Finally, among non-expert adolescent players, having between 2 and 6 years of tennis practice, Cherappurath & Elayaraja (2017) observed an improvement in groundstroke performance (i.e., forehands and backhands) after MI sessions carried out on the tennis court. Indeed, Guillot et al. (2012) recommended realizing MI in ecological conditions (e.g., in sports clothing, with the racket and on the tennis court) in order to facilitate the construction of mental representations of the actions to be carried out. These mental representations, built throughout life on the basis of learning and lived experiences, guide the realization of motor actions (Jeannerod, 1999) and can be reinforced by MI wich allows to increase the amount of practice. Robin and Dominique (2022) suggested integrating MI into learning and training sessions in order to benefit from the additional beneficial effects linked to the combination of real practice and motor imagery. Most research has used MI before real execution (Dominique et al., 2021) and to our knowledge, no studies have used MI after real trials in particular to correct actions inappropriate motor skills leading to an unprovoked fault and simulate a successful tennis action. Indeed, in most studies participants are asked to imagine succeed in the action. The novelty of this research therefore consists of using positive (i.e., success) or negative (i.e., failure) mental images after the negative result of a real action in a training match context specific to each athlete. Given that MI is generated on the basis of mental representations (Jeannerod, 1999) and reinforces them, imagining oneself performing a correctly carried out action helps improve motor learning (Robin & Dominique, 2022; Simonsmeier et al., 2020). However, imagining oneself performing a movement resulting in an error risks impairing motor performance as shown by Woolfolk et al. (1985) on a simple motor task (i.e., golf putting task).

The aim of this study was to evaluate the effects of an MI intervention consisting of imagining oneself succeeding, or failing, a motor action after committing an unprovoked fault, on the performance of groundstrokes (i.e., forehands and backhand) in young tennis players. We hypothesized that participants using success MI would perform better than participants in the control group, while the use of failure MI would lead to a decline in performance. In addition, since imagery has a motivational function, we also hypothesize that success MI practice would promote an increase in the sense of self-efficacy of players who would benefit from this mental practice, while the opposite should be observed for failure MI.

## Methods

### Participants

An a priori power analysis (G*Power 3.1) was used (effect size 0.25; alpha 0.05 and power 0.95) to calculate the total sample size (*N* = 66, with critical F = 3.14 and actual power 0.95).

Sixty-six male players (12.1 years ± 1.89 years) volunteered to participate in this study. The participants had at least 3 years of club practice (M = 3.9; SD = 1.3) and competed at a regional level. The players, as well as their legal guardians, gave their informed consent prior to their inclusion in the study and reported no cognitive impairment. They were randomly distributed, by drawing lots, into the control (*N* = 22), the failure MI (*N* = 22) or the success MI (*N* = 22) groups. This study, carried out in accordance with the ethical standards laid down in the Declaration of Helsinki (1964) and its later amendments, was approved by the local ethics committee of the University (ACTES URp5-4-2023-5).

### Measures and materials

- **Radar gun.** The speed of the balls was measured with a radar gun (Cordless MPH Radar Gun type R1000) and served as a control variable in order to detect a possible speed accuracy trade-off.
- **Imagery ability scale.** This scale makes it possible to evaluate the vividness of the visual images produced by the participants (Kanthack et al., 2016), at the end of each session, using an imagery quality index composed of a Likert scale ranging from 1 (“unclear and not very vivid mental image”) to 6 (“perfectly clear and vivid mental image”) (for a similar procedure, see Robin et al., 2021).
- **Self-efficacy questionnaire.** Participants’ self-efficacy was determined by assessing both the strength and direction of participants’ expectations regarding their subsequent performance on the baseline return task with the ball launcher. All statements followed the format “I think I can make at least x returns on all 30 balls” “no or yes” with “30” being the maximum score. In total, the questionnaire consisted of 6 progressively more difficult statements, with “I think I can make at least 5 returns out of 30 balls” being the first and simplest statement, and “I think I can make 30 returns on 30 balls” being the 6th and most difficult. Participants indicated their sense of self-efficacy by rating their level of confidence in response to the percentage statements, “if yes, I am x% sure” (for a similar procedure, see Kanthack et al., 2016).
- **Ball launcher.** In order to standardize the types of balls (in amplitude, direction and effect) received by the participants, a Lobster Elite TWO type ball launcher (EL02-10) was used during the pre-tests and post-tests.
- **Digital tablet and tracking software.** During the tie-breaks, we recorded the players’ performances using a tablet (Apple Ipad pro 11 512G) equipped with performance collection software (Swingvision). This software makes it possible, in particular, to evaluate the percentage of groundstrokes, the number of rallies, as well as fouls. Two independent expert coaches identified and recorded the number of unprovoked faults during the tie-breaks carried out in the pre- and post-tests.

### Procedure

Before the start of the experimental phase and after signing the consent form, all participants completed the Movement Imagery Questionnaire third French version (MIQ-3f, Robin et al., 2020).

**Fig. 1:**
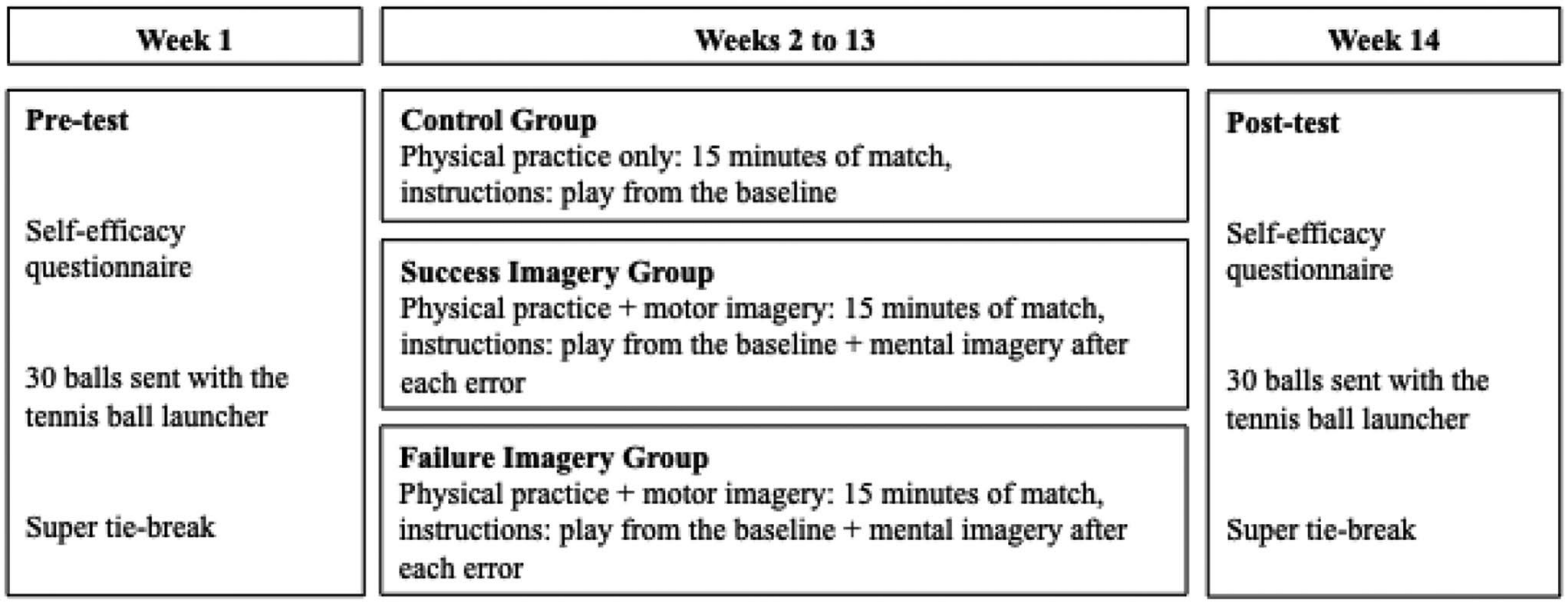
Time course of the experimental design

The 3 experimental phases of this study were spread over 14 weeks (see Fig. 1).

**Fig. 2:**
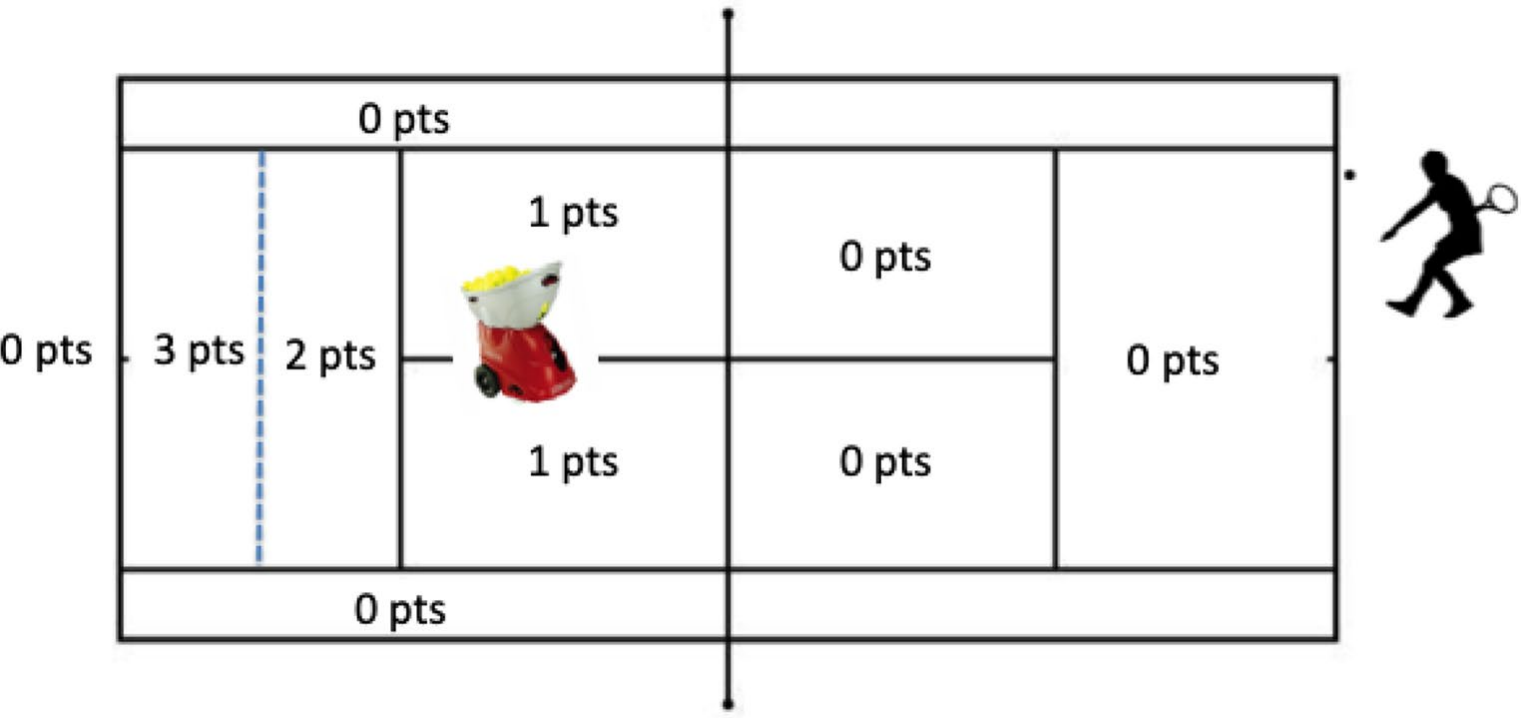
Number of points awarded based on the zone reached by the return

During the first phase (i.e., pre-test), carried out in week 1, the players of the three experimental groups made 6 blocks of 5 baseline shots (forehand and backhand) sent randomly by the ball launcher. It is important to note that the latter made it possible to control the orientation, speed and effects of the balls sent, so that each player received, during each phase, the same types of balls but in a random order. The participants were instructed to make forehand and backhand returns towards the opposite baseline (i.e., a ball rebound zone located close to the baseline, see Fig. 2). Player performance was assessed using different dependent variables. The first was the efficiency score of forehands and backhands, carried out during the pre- and post-tests, ranging from 0 (unforced error) to 3 points for each of the balls located in the baseline area. The second variable was the number of unforced errors committed (i.e., balls in the net or out of bounds) in each block. The speed of the balls, measured using the radar gun, was recorded by the experimenters. Then, the players performed a super tie-break in 10 filmed points, during which the groundstrokes, the number of rallies as well as the number of unprovoked faults were recorded.

The second phase consisted of 12 weekly tennis sessions lasting 35 min. During each of these, the tennis players from the control, failure MI and success MI groups, after a standardized warm-up of 20 min, played with a pair of equivalent level, 15 min of match in tie-break format with engagement in service (2nd lifted ball). The instructions given to all the participants, whatever their group, were to send as many balls as possible into the baseline area. The control group did not receive any particular instructions other than to physically carry out the task. On the other hand, the participants in the MI groups were instructed, after each fault, to imagine themselves using an internal visual modality, that is to say as if they were watching their movements with their own eyes, succeeding in the previous shot with a trajectory ball reaching the baseline (i.e., success MI group) or failing (i.e., failure MI group). At the end of each session, players in the success and failure MI groups were asked to rate the vividness of the visual images they had produced using an imagery quality index (i.e., imagery ability scale; for a similar procedure, see Robin et al., 2021).

The third and final phase, carried out during week 14, served as a post-test. Its composition and procedure were identical to those of the pre-test.

### Data analysis

The data of five of the participants were not taken into account, because the latters were absent during at least one of the experimental sessions (sample used for statistics: control group, *N* = 21; failure MI group, *N* = 19; success MI group, *N* = 21).

Regarding MI ability scores assessed at the end of each session, none of the players in the success and failure MI groups revealed having had difficulties (Robin & Blandin, 2021) in the realization and use of mental images (Mean scores = 4.6; standard deviation = 1.3) between each block carried out during the sessions of the second phase.

For the returns made with the ball launcher, in the pre- and post-tests, the average speed (in kilometre-hour), the number of faults and the precision scores (Fig. 2) were recorded and calculated. Likewise, during tie-breaks, the percentage of rallies (between 5 and 8 balls), the percentage of balls reaching the baseline, and the percentage of unforced errors were calculated using the Swingvision software. ANOVAs were performed, on the basis of these dependent variables, using the following experimental design: 3 independent groups (control vs. success MI vs. failure MI) × 2 tests (pre-test vs. post-test) with repeated measures. Before the ANOVAs, the normality of distribution (Kolmogorov-Smirnov test) and the homogeneity of variances (Levene test) were checked. Newman-Keuls tests were used in post hoc analyses. Alpha was set at *p* = .05 was used and effect sizes (ηp2) are reported for all the analyses that were performed on Statistica (12.0, 64-bit).

## Results

### Self-efficacy

The ANOVA performed on self-efficacy scores revealed main effects of group, *F*(2, 58) = 16.86, *p* < .001, 𝜂_p_^2^ = 0.37, and test, *F*(1, 58) = 14.39, *p* < .001, 𝜂_p_^2^ = 0.20 as well as a significant interaction between group and test, *F*(2, 58) = 37.24, *p* < .001, 𝜂_p_^2^ = 0.35. Post-hoc analysis reveals an improvement in self-efficacy scores from pre-test to post-test (*p* < .001) only for participants in the success MI group who also obtained higher scores than the control and failure MI groups (*p* < .001) at post-test (see Table 1). In addition, participants in the failure MI group decreased their efficacy scores from pre-test to post-test.

**Table 1:** Mean (*standard deviation*) self-efficacy scores for the control, failure imagery and success imagery groups during pre-test and post-test

Groups	Pre-test	Post-test
Control (*N* = 21)	18.91 (*0.49*)	19.04 (*0.63*)
Failure imagery (*N* = 19)	18.68 (*0.52*)	16.84 (*0.52*)
Success imagery (*N* = 21)	18.57 (*0.49*)	23.26 (*0.66*)

### Return speed with the ball launcher

The ANOVA carried out on the average return speeds measured during the pre-test and post-test did not reveal any main effects of group, *F*(2, 58) = 0.33, *p* = .71, 𝜂_p_^2^ = 0.01 or test, *F*(1, 58) = 0.01, *p* = .91, 𝜂_p_^2^ = 0.00 nor significant interaction between group and test, *F*(2, 58) = 0.39, *p* = .67, 𝜂_p_^2^ = 0.01.

## Accuracy scores with the ball launcher

The ANOVA performed on the accuracy scores revealed main effects of group, *F*(2, 58) = 64.90, *p* < .001, 𝜂_p_^2^ = 0.69 and test, *F*(1, 58) = 75.79, *p* < .001, 𝜂_p_^2^ = 0.57 as well as a significant interaction between group and test, *F*(2, 58) = 112.66, *p* < .001, 𝜂_p_^2^ = 0.79.

**Fig. 3:**
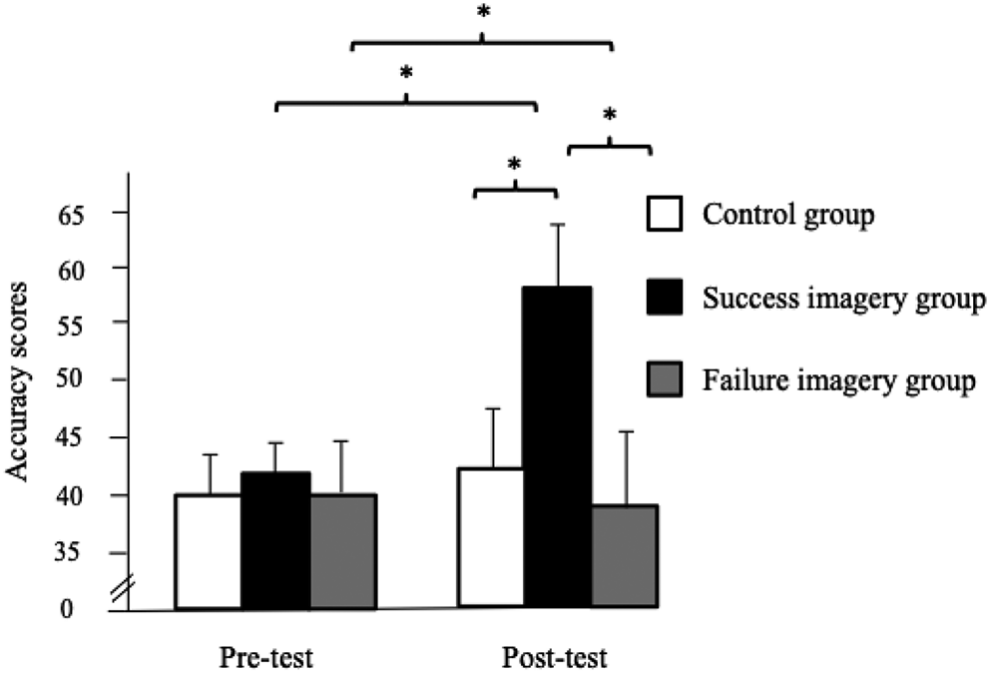
Significant interaction between the group and the test (* *p* < .05) regarding the accuracy scores

Post-hoc analysis reveals a significant improvement (*p* < .001) in accuracy scores from pre-test to post-test for players in the success MI group, who also obtained higher scores than the participants in the control (*p* < .001) and failure MI (*p* < .001) groups at post-test (see Fig. 3). Moreover, participants in the failure MI group decreased their accuracy scores from pre-test to post-test (*p* = .016).

## Number of unforced errors with the ball launcher

**Fig. 4:**
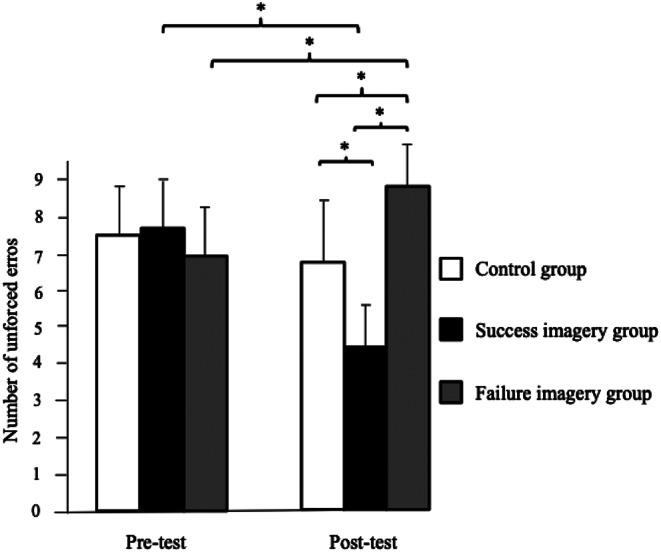
Significant interaction between the group and the test (* *p* < .05) regarding the number of unforced errors

The ANOVA carried out on the faults committed during the pre- and post-tests carried out with the ball launcher revealed a main effect of group, *F*(2, 58) = 11.25, *p* < .001, 𝜂_p_^2^ = 0.28, an absence of main effect of test, *F*(1, 58) = 0.01, *p* = .91, 𝜂_p_^2^ = 0.00 as well as a significant interaction between group and test, *F*(2, 58) = 42.21, *p* < .001, 𝜂_p_^2^ = 0.59. Post-hoc analysis reveals a significant decrement (*p* < .001) in unforced errors from pre-test to post-test for players in the success MI group who also made lower errors than the participants of the control (*p* < .001) and failure MI (*p* < .001) groups at post-test (see Fig. 4). Moreover, participants in the failure MI group increased their unforced errors from pre-test to post-test.

### Percentage of rallies between 5 and 8 balls

The ANOVA carried out on the percentage of rallies during the pre- and post-tests of tie-breaks revealed an absence of main effect of group, *F*(2, 58) = 1.75, *p* = .67, 𝜂_p_^2^ = 0.07, as well as an absence of significant interaction between the group and the test, *F*(2, 58) = 4.72, *p* = .29, 𝜂_p_^2^ = 0.14. However, the ANOVA revealed a may effect of test, *F*(1, 58) = 61.99, *p* < .05, 𝜂_p_^2^ = 0.51. The post-hoc analysis reveals a significant improvement (*p* = .017) in the percentage of exchanges between 5 and 8 from pre-test to post-test.

### Percentage of groundstrokes in tie-breaks

The ANOVA performed on the groundstroke percentage in tie-breaks revealed an absence of main effect of group, *F*(2, 58) = 3.07, *p* = .37, 𝜂_p_^2^ = 0.09, and test *F*(1, 58) = 2.75, *p* = .52, 𝜂_p_^2^ = 0.04. The analyse revealed a significant interaction between the group and the test, *F*(2, 58) = 4.57, *p* = .014, 𝜂_p_^2^ = 0.14. The post-hoc analysis revealed a significant increase (*p* = .003) in groundstroke percentage from pre-test to post-test for the players in the success MI group who also had higher performance than the participants in the control (*p* = .012) and failure (*p* < .001) MI groups at post-test.

### Percentage of unforced errors in tie-breaks

**Fig. 5:**
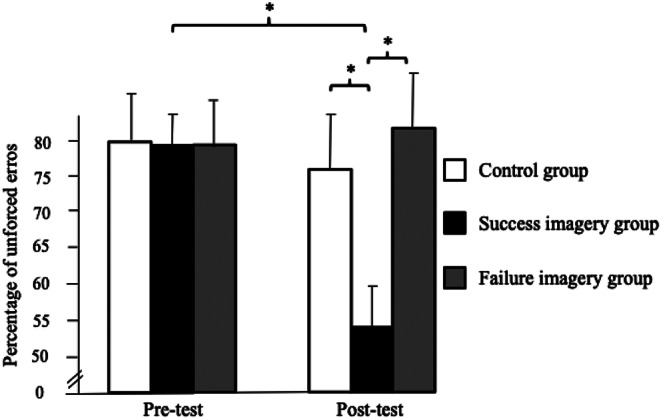
Significant interaction between the group and the test (* *p* < .05) regarding the percentage unforced errors

The ANOVA carried out on the percentage of unforced errors in tie-breaks revealed main effects of group, *F*(2, 58) = 26.78, *p* < .001, 𝜂_p_^2^ = 0.48 and test, *F*(1, 58) = 41.51, *p* < .001, 𝜂_p_^2^ = 0.42 as well as a significant interaction between the group and the test, *F*(2, 58) = 46.96, *p* < .001, 𝜂_p_^2^ = 0.62. The post-hoc analysis reveals a significant decrease (*p* < .001) in the number of unforced errors, from pre-test to the post-test, for the players in the success MI group who also made fewer unforced errors than the participants in the control (*p* < .001) and failure (*p* < .001) MI groups, at post-test (see Fig. 5).

## Discussion

This original study aimed to evaluate the effects of MI consisting of imagining successful, or failing, forehands and backhands, after each unforced errors, on groundstroke performance in tennis players without -experts. The main results of this experiment show that players in the success imagery group improved their performance in the ball-launcher task. Indeed, the latter have significantly increased the precision of their returns towards the back of the tennis court while reducing the number of unforced errors.

In addition, players who benefited from success MI during practice sessions also significantly reduced the number of unforced errors during match situations (i.e., tie-breaks) made in the post-test, in comparison with the pre-test. Finally, and although the difference was not statically different, the players in the success group still obtained a baseline ball percentage 10% higher than that of the control group at the post-test. These results, which validate our first hypothesis, confirm previous work in the literature which has shown beneficial effects of MI on learning and motor performance (Simonsmeier et al., 2020), particularly in racquet sports (Cece et al., 2020) and more specifically in tennis (e.g., Cherappurath et al., 2020; de Sousa Fortes et al., 2019; Dominique et al., 2021; Morais et al., 2019; Robin & Dominique, 2022) with young non-expert players (Cherappurath et al., 2017; Dereceli, 2019; Corrado et al., 2020; Dohme et al., 2020; Guillot et al., 2013; Robin & Dominique, 2022, 2023).

As mentioned by Guillot et al. (2013), the use of MI, in addition to real practice, can promote the improvement of shot precision and reduce the variability of performance in tennis whether in a context of low uncertainty (e.g., serving or with the ball launcher) but also in situations of greater uncertainty (e.g., match or tie-break). The results of this experiment also confirm the positive effects of performing success MI on the field in sports clothing (Guillot et al., 2013), with the racket in hand (Guillot et al., 2012) and integrated into training sessions (Robin & Dominique, 2022; Robin & Dominique, 2022). Indeed, access to mental representations, serving as support for the mental simulation of actions during MI, can be facilitated by the context and the environment in which the imagery is performed (Hall, 2001; Guillot et al., 2013). In addition, we may also envisage that simulating a successful tennis action, after a real error made in a match context specific to each athlete, would make young tennis players even more active and involved in their learning process (Robin & Dominique, 2022). The use of MI could help athletes act as active agents in their own improvement of baseline tennis performance.

However, the results of the current study also revealed that failure motor imagery can have a negative impact on motor performance. This supports learning effect of MI: those who imagined doing errors also learned to do errors and deteriorate their performance following a negative MI intervention (Woolfolk et al., 1985). Indeed, participants who imagined missing forehand and backhand shots made more unforced errors and had lower shot precision than those who performed success MI. Several authors highlighted the importance, for tennis players, of representing images of successful execution through MI (e.g., Dana & Gozalzadeh, 2017; Dereceli, 2019; Robin & Dominique, 2022). In addition, Blankert and Hamstra (2017), defined imagery as “richly imagining carrying out a task successfully” when investigating the effect of MI intervention in service performance. The specific contribution of the current study concerns the fact that we have demonstrated that imagining failing a complex motor action can have a negative impact on motor performance. This is why we exclusively recommend successful action simulation, during MI.

The results obtained in this experiment also show that the players who used MI improved their self-efficacy score, leading to their success in ball launcher task, which validates our second hypothesis. The results of this study confirm those of previous research studies, which show that MI also has a motivational function (Hall et al., 1992; Hardy, 1998; Simonsmeier et al., 2020) and can be used by tennis players and coaches to improve self-confidence and feelings of competence or self-efficacy (Crespo & Reid, 2007; Robin & Dominique, 2022; Weinberg & Jackson, 1990). Since self-efficacy is known to be a powerful “predictor” of sports performance (Feltz et al., 2008), any intervention allowing it to be increased will be useful for coaches and beneficial for players (Weinberg & Jackson, 1990). Given that MI can increase the feeling of competence, self-confidence and motor performance, we encourage players and coaches to use motor imagery, during practice sessions and especially after unforced errors in order to simulate actions corrected by the player himself (Gmamdya et al., 2023).

This study is not without limitation. Although similar to previous research that has used similar procedures (e.g., Cherappurath et al., 2020; Féry & Morizot, 2000; Guillot et al., 2013; Robin & Dominique, 2022), the number of participants per group (*N* = 12) was relatively small, limiting the power of the statistical analyses, which is why the results obtained in this study must be interpreted with caution and should be confirmed with other studies with larger samples. In addition, it is possible that the players in the control group were less motivated than those in the imagery group due to the experimental conditions, which may also have prevented them from realizing MI after certain errors as some players seem to do on tennis court. The use of a scale a questionnaire on self-efficacy, solely focused on ball throwing performance, can be considered a limitation. Finally, the participants who had to perform the MI during the acquisition phase, did so after each of their own faults but the amount of mental practice was not noted, which may represent another limitation of this study.

As an extension of this study, it would be interesting to test whether the performance improvements would be greater when the players benefit from feedback from the coach, concerning their previous fault, before carrying out the MI as observed by Robin et al. (2020) in a football accuracy task. Finally, while in this study the internal visual imagery modality was imposed during MI practice, on the one hand it is possible that the results could be modulated by the use of other MI modalities (e.g., external visual or kinesthetic) as recently shown by Dominique et al. (2021) on tennis serve, and on the other hand that the use of multimodal imagery of action (Krüger et al., 2024) could optimize the effect of MI. Additional research work will soon be carried out in our laboratory to test these hypotheses.

## Conclusion

The main results of this study show that 12 weeks of practice, during which the young tennis players were instructed to imagine corrected forehands and backhands, after each unforced error, increases the feeling of efficacy, improves precision of groundstrokes and reduce the number of unforced errors, whether with the ball launcher or in a match situation. These results confirm those of previous research studies having shown the importance of using MI in the field of sports performance. It is also important to emphasize that the results of the current study also showed the deleterious effects of failure MI on background stroke performance. These results lead us to recommend that players and coaches use success motor imagery during their training sessions in order to improve baseline performance, as well as confidence. In an applied manner, the beneficial effects of motor imagery integrated into practice sessions and carried out after each unforced error lead us to suggest its use in training session to improve the quality of the baseline play in young non-experts tennis players.

## Data Availability

We have full control of all primary data and we agree to allow the journal to review our data if requested.
